# Multimodal imaging of sarcoid choroidal granulomas

**DOI:** 10.1186/1869-5760-3-58

**Published:** 2013-08-23

**Authors:** Yasha S Modi, Aliza Epstein, Swetangi Bhaleeya, J William Harbour, Thomas Albini

**Affiliations:** 1 Department of Ophthalmology, Bascom Palmer Eye Institute, Miller School of Medicine, University of Miami, Miami, FL 33136, USA; 2 Department of Ophthalmology, New York Presbyterian Hospital, Weill Cornell Medical College, Cornell University, New York, NY 10021, USA

**Keywords:** Choroidal granuloma, Sarcoidosis, Imaging, Enhanced-depth imaging optical coherence tomography, Indocyanine green

## Abstract

**Background:**

Enhanced-depth imaging optical coherence tomography (EDI-OCT) provides high-resolution imaging of the choroid. Herein, we report multimodal imaging, including EDI-OCT, of a case of sarcoid choroidal granulomas.

**Findings:**

A 63-year-old female with biopsy-supported sarcoidosis presented with unilateral multifocal choroidal granulomas. Enhanced-depth imaging optical coherence tomography (EDI-OCT) demonstrated a homogenous hyporeflective choroidal lesion with choriocapillaris thinning and sparing of the surrounding choroid. The patient was started on oral steroids with a weekly taper schedule. Within 5 weeks, the choroidal lesions had clinically resolved with return of normal-appearing choroidal architecture on EDI-OCT. Indocyanine green angiography, however, demonstrated hypofluoresence at the sites of choroidal granulomas 11 months after the clinical resolution, suggesting a longstanding choroidal perfusion deficit undetected by OCT.

**Conclusions:**

Choroidal imaging via EDI-OCT provides detailed morphologic information of sarcoid granulomas and can accurately demonstrate structural resolution of the lesions.

## Findings

This is a longitudinal case report of a patient with unilateral multifocal choroidal granulomas in the setting of systemic sarcoidosis. Multimodal imaging was used to aid in diagnosis and follow through resolution.

### Introduction

Choroidal granulomas in the absence of anterior uveitis are a rare but well-recognized manifestation of sarcoidosis, occurring in approximately 5% of patients with ocular sarcoidosis [1]. Accurate imaging to correctly differentiate granulomas from amelanotic melanomas and choroidal metastasis is critical prior to developing a treatment plan. Enhanced-depth imaging optical coherence tomography (EDI-OCT), a novel imaging modality that provides excellent morphologic details of the choroid, has recently been used to evaluate choroidal pathology in various disease states including central serous chorioretinopathy [2], myopia [3], macular degeneration [4], choroidal tumors [5], and a *Toxocara* optic nerve granuloma [6]. However, EDI-OCT imaging of choroidal granulomas in the setting of sarcoidosis has not been previously reported.

Herein, we report a case of unilateral multifocal choroidal sarcoid granulomas that underwent multimodal imaging including EDI-OCT, fluorescein angiography (FA), and indocyanine green angiography (ICG).

### Report of a case

A 63-year-old black female with recently diagnosed biopsy-supported systemic sarcoidosis involving the lungs and skin presented with an insidious, painless decrease in vision of the right eye with metamorphopsia over a 3-month period. A recent medical evaluation revealed lymphadenopathy on chest computed tomography, erythema nodosum of the lower extremities, and a skin biopsy demonstrating noncaseating granulomas consistent with sarcoidosis. She had not started any systemic immunosuppression at the time of presentation. The patient consented for this case to be presented.

**Figure 1:**
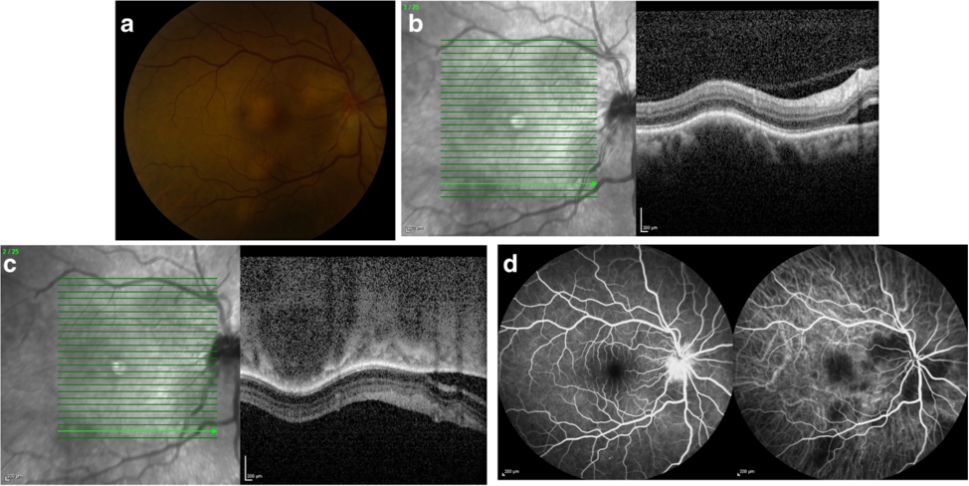
**Color photo, EDI-OCT, FA, and ICG images of the right eye on presentation.** Color photograph of the fundus of the right eye, showing multifocal deep, creamy, elevated lesions **(a)**. Standard OCT demonstrates a choroidal lesion with overlying elevation of the RPE and retina and subretinal fluid adjacent to the peripapillary lesion **(b)**. EDI-OCT demonstrates the hyporeflective and well-circumscribed choroidal lesion, overlying choriocapillaris thinning, and its posterior margin **(c)**. FA **(d**, left**)** demonstrates disc leakage, and ICG **(d**, right**)** demonstrates hypofluorescence corresponding to the sites of choroidal granulomas.

On examination, best-corrected visual acuity (BCVA) was 20/30 and 20/20 in the right eye and left eye, respectively. Anterior segment examination of both eyes revealed a quiet anterior chamber without cells or flare. The posterior segment of the right eye (Figure 1a) was notable for the absence of vitritis and multiple deep, focal, elevated posterior creamy lesions and more diffuse peripapillary focal creamy lesions obscuring the disc margins. There was associated subretinal fluid (SRF) adjacent to the peripapillary lesions. Posterior segment examination of the left eye was normal without evidence of vitritis or choroidal lesions. Spectral domain optical coherence tomography (SD-OCT) (Figure 1b) over a choroidal lesion showed focal elevation of the retinal pigment epithelium (RPE) and retina with shadowing deep to the lesion and adjacent SRF emanating from the peripapillary granulomas. EDI-OCT (Figure 1c) through the same area better characterizes the homogenous hyporeflective lesion and its posterior margin, thinning of the overlying choriocapillaris, and surrounding uninvolved choroidal architecture. Early FA/ICG images (Figure 1d) revealed disc leakage and hypofluoresence on ICG corresponding to the sites of the choroidal lesions.

**Figure 2:**
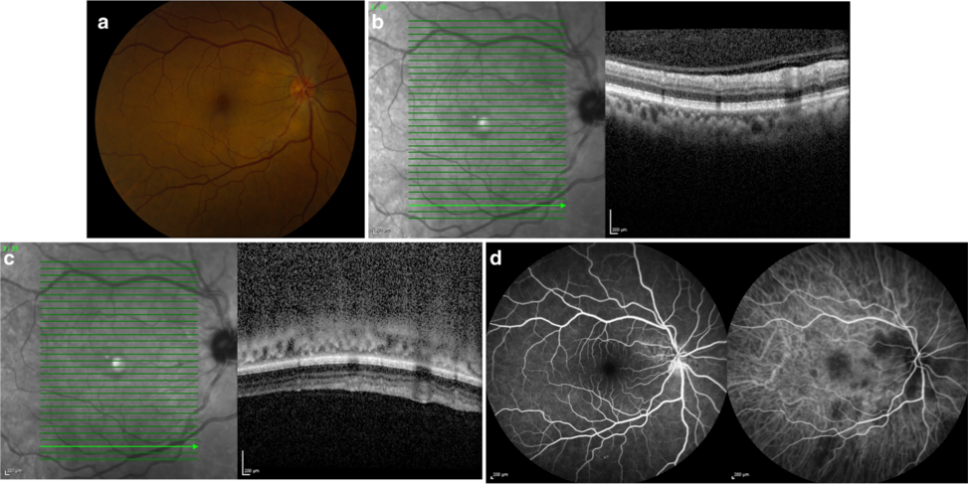
**Color photo, EDI-OCT, FA, and ICG images of the right eye 5 weeks after treatment initiation.** A color fundus photograph of the right eye demonstrates resolution of the elevated lesions with resolution of the subretinal fluid. There is mottling of the RPE over the lesions sites **(a)**. Standard OCT **(b)** and EDI-OCT **(c)** demonstrate resolution of the choroidal lesion. FA **(d,** left**)** demonstrates resolution of disc leakage, and ICG **(d,** right**)** demonstrates persistent hypofluorescence corresponding to the sites of prior choroidal granulomas.

Given the medical and ophthalmic work-up favoring sarcoidosis with multifocal choroidal granulomas, the patient was started on 60 mg of oral prednisone with a weekly taper of 10-mg decrements. Five weeks after presentation, the patient’s metamorphopsia had resolved, with BCVA improving to 20/25. The elevated choroidal lesions had resolved along with the SRF, but there was mild hypopigmentation over the lesion sites (Figure 2a). SD-OCT (Figure 2b) through the same area demonstrated resolution of the choroidal granuloma. EDI-OCT (Figure 2c) demonstrated the posterior margin of the choroid, absence of the hyporeflective lesion, and normal architecture. However, ICG (Figure 2d) demonstrated persistent choroidal hypofluoresence corresponding to the original lesion sites. The optic disc leakage had resolved, however (Figure 2d).

**Figure 3:**
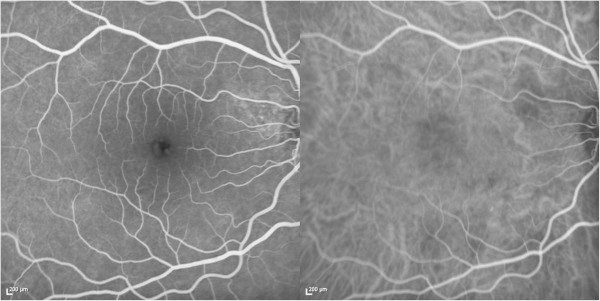
**FA and ICG 11 months after treatment initiation.** FA (left) demonstrates no disc leakage. ICG (right) demonstrates mild hypofluorescence corresponding to the sites of prior choroidal granulomas.

The patient refused methotrexate as a steroid-sparing alternative and was subsequently maintained on 5 mg of prednisone daily with no recurrence of the choroidal granulomas. Twelve months after presentation, the choroidal granulomas did not recur. Repeat ICG (Figure 3) demonstrated significantly improved but still present hypofluorescence.

## Discussion

Choroidal granulomas manifesting as the sole lesion in ocular sarcoidosis has been previously described [1,7-10]. Desai et al. reported on the largest case series of choroidal granulomas in nine patients with biopsy-proven sarcoidosis [1]. Eight patients presented with solitary choroidal granulomas, and one patient presented with multifocal involvement. All the patients were started on oral prednisone with nine out of nine eyes demonstrating a reduction in size of the granuloma and two out of nine eyes demonstrating complete resolution. Five of the eyes experienced a recurrence, however, with an average time to recurrence of 7.6 months [1].

EDI-OCT imaging of the choroid, in this case, provided accurate morphologic characterization of the choroidal granuloma and its subsequent resolution. Salman et al. previously characterized tuberculous granulomas via a Stratus OCT (Carl Zeiss Meditec Inc., Dublin, CA, USA), which demonstrated the relationship of the anterior granuloma with the RPE and retina [11]. However, the morphology and the posterior margin were not well characterized. The resolving ability of EDI-OCT for choroidal lesions has been reported as 1 mm in depth, allowing for accurate qualitative and quantitative evaluation of small choroidal lesions too small to be characterized via ultrasound [12]. Additionally, EDI-OCT may provide the ability to detect interval change of the granuloma size in response to treatment and, potentially, early recurrence.

After 1 month of treatment on oral steroids, the granulomas in this patient clinically disappeared with residual RPE mottling and hypopigmentation over the sites of prior granulomas. Repeat imaging via EDI-OCT demonstrated resolution of the lesion with an apparent return of normal choroidal architecture. The ICG, however, continued to demonstrate hypofluorescence in the involved sites 11 months after clinical resolution. It is possible that this may represent a longstanding choroidal perfusion deficit that cannot be resolved via OCT. Subsequent EDI-OCT has not demonstrated early recurrence of these lesions, and the patient will continue maintenance therapy with low-dose steroids.

In conclusion, choroidal imaging via EDI-OCT provides detailed morphologic information of sarcoid granulomas and can accurately demonstrate structural resolution of the lesions. Further studies are required to identify defining features that may aid in differentiation from other inflammatory and neoplastic processes.

## Abbreviations

BCVA: Best-corrected visual acuity; EDI-OCT: Enhanced-depth imaging optical coherence tomography; FA: Fluorescein angiography; ICG: Indocyanine green angiography; RPE: Retinal pigment epithelium; SD-OCT: Spectral domain optical coherence tomography; SRF: Subretinal fluid.

## Competing interests

The authors declare that they have no competing interests.

## Authors’ contributions

YSM participated in the conception of this report, researched the topic, primarily wrote the manuscript, participated in image selection, and edited the manuscript. AE helped write and edit the manuscript, researched the topic, and primarily assembled the figures. SB clinically managed the patient and helped write and edit the manuscript. JWH participated in the conception of this report and edited the manuscript. TA clinically managed the patient, participated in the conception of this report, and edited the manuscript. All authors read and approved the final manuscript.

## Authors’ information

YSM is a second year ophthalmology resident at the Bascom Palmer Eye Institute. He has a clinical and academic interest in uveitis, choroidal tumors, and vitreoretinal surgery. AE is a third year medical student at the University of Miami School of Medicine. She is interested in ophthalmology. SB was a former Uveitis fellow at the Bascom Palmer Eye Institute under the auspices of Dr. Janet Davis and Dr. Thomas Albini; she is currently pursuing a vitreoretinal fellowship at Cornell University. JWH is a professor of Ophthalmology at the Bascom Palmer Eye Institute. His clinical interests include ocular oncology and vitreoretinal diseases and surgery. TA is an associate professor of Ophthalmology at the Bascom Palmer Eye Institute. His clinical interests include uveitis and vitreoretinal diseases and surgery.
